# The complete mitochondrial genome of the endangered great hammerhead shark, *Sphyrna mokarran*

**DOI:** 10.1080/23802359.2017.1318682

**Published:** 2017-04-25

**Authors:** Cassandra L. Ruck, Nicholas Marra, Mahmood S. Shivji, Michael J. Stanhope

**Affiliations:** a Save Our Seas Shark Research Center and Guy Harvey Research Institute, Nova Southeastern University, Dania Beach, FL, USA;; b Department of Population Medicine and Diagnostic Sciences, College of Veterinary Medicine, Cornell University, Ithaca, NY, USA

**Keywords:** *Sphyrna mokarran*, mitochondrial genome, great hammerhead

## Abstract

We present the first mitochondrial genome sequence of the great hammerhead shark, *Sphyrna mokarran*. This species is of considerable conservation concern throughout its global distribution, and currently listed as Endangered on the IUCN Red List. The mitochondrial genome is 16,719 bp in length with 13 protein-coding genes, 22 tRNA genes, 2 rRNA genes and a non-coding control region. The gene arrangement is congruent with other shark and most vertebrate species. This *S. mokarran* mitogenome provides a genomic resource for assisting with population studies and conservation efforts for this highly depleted species.

The great hammerhead shark, *Sphyrna mokarran* (Sphyrnidae), is an elasmobranch of considerable conservation concern (Endangered: International Union for the Conservation of Nature (IUCN) Red List) (Denham et al. 2007). The slow life-history traits of this shark coupled with commercial overexploitation for its high value fins has resulted in substantial population declines throughout much of its distribution. Due to the slower evolutionary rate of mitochondrial genes in elasmobranchs compared to other vertebrates, complete mitochondrial genome sequences can offer greater resolution than single gene studies when examining the population structure and phylogeny of elasmobranchs (Feutry et al. 2014; Martin et al. 1992). This higher resolution can be critical in elucidating evolutionary significant units for management and conservation purposes (Feutry et al. 2015). Here we present the first characterization of the whole mitochondrial genome of the great hammerhead shark to assist in future population genetic, conservation and phylogenetic assessments.

The specimen sequenced was a male shark sampled in 2010 off the Florida Keys, USA (geospatial coordinates: 24.36667, −82.41666). A fin clip sample (accession number OC-226) is stored in 100% ethanol at Nova Southeastern University, College of Natural Sciences and Oceanography. DNA was extracted from the fin clip using a QIAGEN DNeasy Blood & Tissue Extraction Kit (Qiagen Inc., Valencia, CA) and amplified in four fragments via long PCR using KAPA HiFi HotStart ReadMix PCR (Kapa Biosystems, Boston, MA). Amplicons were purified with QIAGEN QIAquick Gel Extraction Kit and Agencourt^®^ AMPure XP beads (Beckman Coulter Life Sciences, Indianapolis, IN). Libraries were prepared for sequencing on an Illumina MiSeq system with paired-end reads (2x250 bp) using a Nextera XT DNA Library Preparation Kit. Fastq files were trimmed using the Minoche protocol (Eren et al. 2013). A *de novo* assembly was created with Velvet 1.1 (Zerbino 2010), and the single ambiguity seen resolved by performing a subsequent assembly with Bowtie2 (Langmead & Salzberg 2012) using the consensus sequence from Velvet as the reference index. This final assembly was annotated using MitoAnnotator (Iwasaki et al. 2013).

The mitochondrial genome of the great hammerhead shark (gb: KY464952) is 16,719 bp long containing 13 protein-coding, 22 tRNA, and 2 rRNA genes, and 1 non-coding control region. The nucleotide base composition is: 31.6% A, 25.6% C, 13.0% G, 29.8% T. The *COI* gene began with the alternative start codon GTG. The *COII* and *ND4* genes contained incomplete stop codons. A MUSCLE alignment was performed in Geneious^®^ 7.1.9 (http://geneious.com, Kearse et al. 2012) with the four other published Sphyrnidae mitochondrial genomes (*Eusphyra* blochii: NC_031812.1, *Sphyrna lewini* RefSeq: NC_022679.1, *Sphyrna tiburo* RefSeq: NC_028508.1, *Sphyrna zygaena* RefSeq: NC_025778.1), using the blacktip reef shark, *Carcharhinus melanopterus*, mitochondrial genome as the outgroup (RefSeq: NC_024284.1). The order of genes on the great hammerhead mitochondrial genome is congruent with the other Sphyrnidae sharks. A Bayesian tree (Figure 1) was created with default parameters in MrBayes 3.2 (Huelsenbeck & Ronquist 2001; Ronquist & Huelsenbeck 2003) and the best substitution model (GTR + I) determined by the Bayesian Information Criterion in JModelTest2.1.7 (Darriba et al. 2012).

**Figure 1.**
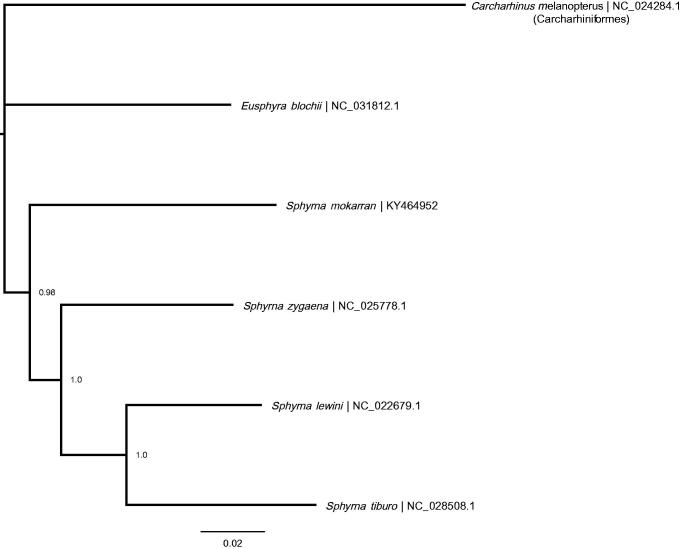
Bayesian tree depicting the five available Sphyrnidae mitogenomes with a Carcharhiniformes shark species (*Carcharhinus melanopterus*) as the outgroup. NCBI RefSeq numbers, branch lengths and posterior probability of the clades are shown.

## References

[CIT0014] Darriba D, Taboada GL, Doallo R, Posada D. 2012 jModelTest 2: more models, new heuristics and high-performance computing. Nat Methods. 9:772. 10.1038/nmeth.2109 PMC4594756 22847109

[CIT0015] Denham J, Stevens J, Simpfendorfer CA, Heupel MR, Cliff G, Morgan A, Graham R, Ducrocq M, Dulvy ND, Seisay M, et al. 2007 Sphyrna mokarran. The IUCN Red List of Threatened Species. 2007:e.T39386A10191938.

[CIT0002] Eren AM, Vineis JH, Morrison HG, Sogin ML. 2013 A filtering method to generate high quality short reads using Illumina paired-end technology. PLoS One. 8:e66643. 23799126 10.1371/journal.pone.0066643 PMC3684618

[CIT0003] Feutry P, Kyne PM, Pillans RD, Chen X, Marthick JR, Morgan DL, Grewe PM. 2015 Whole mitogenome sequencing refines population structure of the Critically Endangered sawfish Pristis pristis. Mar Ecol Prog Ser. 533:237–244.

[CIT0004] Feutry P, Kyne PM, Pillans RD, Chen X, Naylor GJ, Grewe PM. 2014 Mitogenomics of the Speartooth Shark challenges ten years of control region sequencing. BMC Evol Biol. 14:232. 25406508 10.1186/s12862-014-0232-x PMC4245800

[CIT0005] Huelsenbeck JP, Ronquist F. 2001 MRBAYES: Bayesian inference of phylogenetic trees. Bioinformatics. 17:754–755. 11524383 10.1093/bioinformatics/17.8.754

[CIT0006] Iwasaki W, Fukunaga T, Isagozawa R, Yamada K, Maeda Y, Satoh TP, Sado T, Mabuchi K, Takeshima H, Miya M. 2013 MitoFish and MitoAnnotator: a mitochondrial genome database of fish with an accurate and automatic annotation pipeline. Mol Biol Evol. 30:2531–2540. 23955518 10.1093/molbev/mst141 PMC3808866

[CIT0007] Kearse M, Moir R, Wilson A, Stones-Havas S, Cheung M, Sturrock S, Buxton S, Cooper A, Markowitz S, Duran C, et al 2012 Geneious Basic: an integrated and extendable desktop software platform for the organization and analysis of sequence data. Bioinformatics. 28:1647–1649. 22543367 10.1093/bioinformatics/bts199 PMC3371832

[CIT0008] Langmead B, Salzberg SL. 2012 Fast gapped-read alignment with Bowtie 2. Nat Methods. 9:357–359. 22388286 10.1038/nmeth.1923 PMC3322381

[CIT0009] Martin AP, Naylor GJP, Palumbi SR. 1992 Rates of mitochondrial DNA evolution in sharks are slow compared with mammals. Nature. 357:153–155. 1579163 10.1038/357153a0

[CIT0010] Ronquist F, Huelsenbeck JP. 2003 MRBAYES 3: Bayesian phylogenetic inference under mixed models. Bioinformatics. 19:1572–1574. 12912839 10.1093/bioinformatics/btg180

[CIT0001] Sphyrna mokarran . [cited 2017 Apr 19]. Available from: 10.2305/IUCN.UK.2007.RLTS.T39386A10191938.en

[CIT0011] Zerbino DR. 2010 Using the velvet de novo assembler for short‐read sequencing technologies. Curr Protocols Bioinform. 31:11.5.1–11.5.12. 10.1002/0471250953.bi1105s31 PMC2952100 20836074

